# Supplementary material for: Development of an LNP-Encapsulated mRNA-RBD Vaccine against SARS-CoV-2 and Its Variants
Source: Pharmaceutics. 2022 May 20;14(5):1101. doi: 10.3390/pharmaceutics14051101 (PMC9143166; doi:10.3390/pharmaceutics14051101)
Supplement: Supplementary file 1 [file pharmaceutics-14-01101-s001.zip › pharmaceutics-1630686-supplementary.pdf]

Amino acid sequence of RBD

MDAMKRGLCCVLLLCGAVFVSARVQPTESIVRFPNITNLCPFGEVFNATRFASVYAWN
RKRISNCVADYSVLYNSASFSTFKCYGVSPTKLNDLCFTNVYADSFVIRGDEVRQIAP
GQTGKIADYNYKLPDDFTGCVIAWNSNNLDSKVGGNYNYLYRLFRKSNLKPFERDIS
TEIYQAGSTPCNGVEGFNCYFPLQSYGFQPTNGVGYQPYRVVVLSFELLHAPATVCGP
KKSTNLVKNKCVNF
